# Association Between Gastroesophageal Reflux Disease and Periodontitis: A Longitudinal Follow-Up Study from the Korean National Health Screening Cohort

**DOI:** 10.3390/biomedicines12112491

**Published:** 2024-10-30

**Authors:** Na-Eun Lee, Kyeong Min Han, Dae Myoung Yoo, Ho Suk Kang, Ji Hee Kim, Joo-Hee Kim, Woo Jin Bang, Hyo Geun Choi, Ha Young Park, Nan Young Kim, Mi Jung Kwon

**Affiliations:** 1 Hallym Data Science Laboratory, Hallym University College of Medicine, Anyang 14068, Republic of Korea; intriguingly@hallym.ac.kr (N.-E.L.); km.han@hallym.ac.kr (K.M.H.); ydm@hallym.ac.kr (D.M.Y.); 2 Laboratory of Brain and Cognitive Sciences for Convergence Medicine, Hallym University College of Medicine, Anyang 14068, Republic of Korea; 3 Division of Gastroenterology, Department of Internal Medicine, Hallym University Sacred Heart Hospital, Hallym University College of Medicine, Anyang 14068, Republic of Korea; hskang76@hallym.or.kr; 4 Department of Neurosurgery, Hallym University Sacred Heart Hospital, Hallym University College of Medicine, Anyang 14068, Republic of Korea; kimjihee.ns@gmail.com; 5 Division of Pulmonary, Allergy, and Critical Care Medicine, Department of Medicine, Hallym University Sacred Heart Hospital, Hallym University College of Medicine, Anyang 14068, Republic of Korea; luxjhee@gmail.com; 6 Department of Urology, Hallym University Sacred Heart Hospital, Hallym University College of Medicine, Anyang 14068, Republic of Korea; yybbang@hallym.or.kr; 7 Suseo Seoul E.N.T. Clinic, 10, Bamgogae-ro 1-gil, Gangnam-gu, Seoul 06349, Republic of Korea; mdanalytics@naver.com; 8 Department of Pathology, Busan Paik Hospital, Inje University College of Medicine, Busan 47392, Republic of Korea; hy08.park@gmail.com; 9 Hallym Institute of Translational Genomics and Bioinformatics, Hallym University Medical Center, Anyang 14068, Republic of Korea; honeyny78@gmail.com; 10 Department of Pathology, Hallym University Sacred Heart Hospital, Hallym University College of Medicine, Anyang 14068, Republic of Korea

**Keywords:** periodontitis, gastroesophageal reflux disease, longitudinal follow-up study, national health screening cohort data

## Abstract

Background: Gastroesophageal reflux disease (GERD) and periodontitis are common conditions in older adults that can lead to serious complications, gaining public health attention. Although GERD and periodontitis share common risk factors, such as age, lifestyle habits, and socioeconomic status, large-scale studies examining their specific relationship are limited. Methods: This study aimed to assess the association between GERD and the likelihood of developing periodontitis using a national validated cohort data from Korea. Data were drawn from the Korean National Health Insurance Service Health Screening Cohort (2004–2019) using the International Classification of Diseases, 10th Revision, Clinical Modification (ICD-10-CM) diagnostic codes. The study included 16,744 GERD patients and 66,976 matched controls (matched 1:4 by age, sex, income, and residence). Cox proportional hazard models estimated hazard ratios (HRs) for periodontitis, adjusting for various factors, with subgroup analyses based on age, income, and other covariates. Results: The overall incidence of periodontitis was similar between the GERD and control groups, with no significant difference in the adjusted hazard ratios (aHR = 1.00, 95% CI 0.99–1.02, *p* = 0.625). However, subgroup analyses in the GERD group showed a slightly increased likelihood of periodontitis in individuals over 60 years old (aHR = 1.03, 95% CI 1.00–1.06, *p* = 0.050) and those in lower-income brackets (aHR = 1.03, 95% CI 1.01–1.06, *p* = 0.023). Conclusions: In summary, while GERD was not associated with an increased overall probability of periodontitis, age and socioeconomic factors may influence the likelihood of periodontitis development in certain GERD patients. These findings may highlight the need for targeted preventive strategies and closer oral health monitoring in older and lower-income populations with GERD.

## 1. Introduction

Gastroesophageal reflux disease (GERD) is a condition where stomach contents flow back into the esophagus, causing symptoms like acid regurgitation and heartburn [1]. It is the most common esophageal disorder worldwide, with a prevalence of 13.98%, though rates vary across regions [2,3]. The increasing prevalence of GERD is associated with aging, male sex, race, analgesic use, diet, smoking, alcohol, genetics, obesity, and low physical activity [4,5,6]. In East Asia, including Korea, where the prevalence is relatively lower (4.6–7.3%), rates are increasing due to changing lifestyle and dietary habits [2]. Prolonged GERD can cause complications like esophagitis, peptic strictures, Barrett’s esophagus, esophageal adenocarcinoma [1], and extraesophageal issues, such as asthma, laryngitis, pneumonia, and oral health issues, like dental erosion and periodontitis [6]. Some studies suggest that the acidic environment caused by GERD may directly damage oral tissues, increasing susceptibility to periodontal infections [7,8]. A small retrospective study of 116 consecutive GERD patients found that 25.5% had periodontitis and 44% experienced dental erosion [9]. Dzhamaldinova et al. [10] conducted the first study in 2010 on the association between GERD and periodontitis, observing a pathogenic link between the two conditions. However, large-scale studies investigating the specific relationship between GERD and periodontitis remain limited.

Periodontitis is a common inflammatory disease that damages the structures supporting teeth, leading to gum and bone loss and contributing to tooth loss in adults [11,12]. Approximately 11.2% of adults suffer from severe and advanced periodontitis [12], which significantly impacts their quality of life [13,14]. In Korea, periodontitis accounts for 23.4% of periodontal disease cases and is the second leading cause of outpatient visits [15]. Given the rising prevalence of both GERD and periodontitis in Korea, particularly among older adults, the potential comorbidity between these conditions has become a growing concern. Periodontitis affects 85.5% of individuals aged 60–69 [15], while GERD symptoms are more common in those aged 50 and older [16]. Understanding the link between GERD and periodontitis is crucial, especially considering the global rise in GERD incidence and periodontitis’ established systemic effects [12]. The 2018 classification by the European Federation of Periodontology (EFP) and the American Academy of Periodontology (AAP) emphasizes the systemic implications of periodontitis [17,18], linking it to conditions like cardiovascular disease [19,20], diabetes [21], kidney disorders [22], and neurodegenerative diseases [23].

Despite this, only six epidemiological studies have explored the association between GERD and periodontitis [9,10,24,25,26,27], with most showing a positive correlation [9,10,24,25,26], while one found no significant link [27]. These studies were largely based on small samples, single-center designs, and inconsistent demographic data, often neglecting confounding factors like socioeconomic status and comorbid conditions [9,10,25,26,27]. To address these limitations, further research using large, well-matched national cohort data is needed. Given the shared risk factors and potential reciprocal relationship between GERD and periodontitis, it is crucial to conduct a longitudinal follow-up study that considers common confounding factors to clarify the association between GERD and the risk of developing periodontitis.

The aim of this study is to investigate the potential association between GERD and the risk of developing periodontitis with long-term data. Using a large, validated national cohort from South Korea’s public healthcare system, we examine whether GERD increases the likelihood of periodontitis and how individual factors, such as demographics, socioeconomic status, and comorbidities, may influence this relationship.

## 2. Materials and Methods

### 2.1. Data Source

In this study, the Korean National Health Insurance Service Health Screening Cohort (KNHIS-HSC) was utilized as the primary data source. This cohort is anonymized for research purposes, ensuring the protection of personal information by preventing the identification of individuals. South Korea’s National Health Insurance ensures broad representation by covering over 98% of the population through mandatory health insurance policies. It includes individuals who have participated in government-sponsored health screening programs. As of 2002, out of 5.15 million health insurance qualifiers aged 40 to 79, 514,866 individuals were randomly sampled and tracked until 2019. With South Korea’s population estimated at around 55 million, this cohort accounts for roughly 1% of the entire populace [28]. This represents approximately 10% of the total population of health insurance qualifiers in the group. This cohort is continuously managed and regularly updated. The KNHIS-HSC registry relies on diagnostic codes from the International Classification of Diseases, 10th Revision, Clinical Modification (ICD-10-CM), and has been detailed in earlier descriptions [28]. The study was authorized by the Hallym University Ethics Committee (IRB No. 2019-10-023) and adhered to all standards and regulations of the Institutional Review Board. Since the research involved reanalysis of deidentified prospective data, signed consent from participants was not required.

### 2.2. Gastroesophageal Reflux Disease (Exposure)

We included participants diagnosed with GERD with esophagitis (ICD-10: K210) or without esophagitis (ICD-10: K219) between 2002 and 2008. GERD can be classified into three phenotypes: nonerosive reflux disease, erosive esophagitis, and Barrett’s esophagus [29]. Barrett’s esophagus, a form of gastric metaplasia, is typically considered GERD without esophagitis [29]. ICD-10 code K210 is used for GERD with esophagitis, while K219 is for GERD without esophagitis [29,30]. A 2-week trial of proton pump inhibitors (PPI) is recommended for diagnosing typical GERD, and GERD is defined as persistent symptoms after at least 8 weeks of treatment with acid-suppressive agents [29,30]. For our study, we selected patients diagnosed with GERD more than three times and treated with PPI for at least eight weeks [29,30].

### 2.3. Periodontitis (Outcome)

Only individuals who had been treated for periodontitis by dentists and received a confirmed diagnosis using the specific ICD-10 code K05.3 were included to enhance the precision of our analysis and reduce the likelihood of false-positive cases. We documented the frequency of clinic or hospital visits related to periodontitis treatment on an annual basis [23].

### 2.4. Study and Control Group Participants

To explore the potential relationship between GERD and the incidence of periodontitis, we utilized a longitudinal follow-up study design. From the Korean National Health Insurance Service Health Screening Cohort (KNHIS-HSC), which includes 514,866 individuals aged 40 and above, we identified 36,542 individuals who met the specified inclusion criteria for GERD between 2002 and 2008.

The index date of every GERD patient was established as the day when the ICD-10 code for GERD (K210 or K219) was electronically assigned to participants in health insurance claims datasets. To ensure the inclusion of only first-time GERD diagnoses, we excluded 5867 patients diagnosed within the first two years (2002 and 2003) as a washout period, to avoid the potential inclusion of pre-existing GERD cases prior to the index date. Additionally, GERD participants lacking records of body mass index (BMI) or fasting blood glucose were also excluded (n = 4), along with those with any prior medical history of periodontitis before the index date (n = 13,925).

For the control group, exclusions were based on the ICD-10 codes K210 or K219: those diagnosed ≤ 2 times or ≥3 times without a prescription for GERD medication were removed (n = 6562), using a random selection process to minimize selection bias.

GERD participants diagnosed between 2004 and 2008 were matched with control participants from the same period in a 1:4 ratio based on age, sex, income, and region of residence. This procedure resulted in the exclusion of 404,786 control participants and 2 GERD cases who could not be matched, leaving 16,744 GERD individuals successfully paired with 66,976 controls. We then tracked occurrences of periodontitis from each participant’s index date until 31 December 2019, for further analysis.

**Figure 1:**
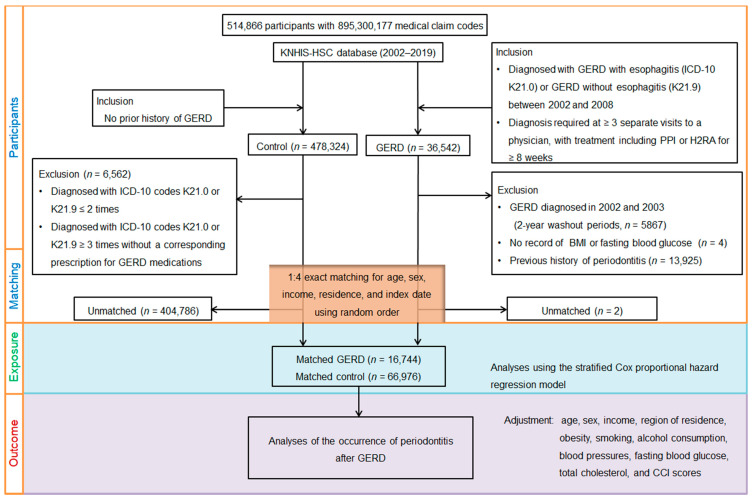
A flowchart illustrating the selection and matching process of patients and the control group from the Korean National Health Insurance Service Health Screening Cohort (KNHIS-HSC). The process involved several steps including initial screening, application of inclusion and exclusion criteria, and final matching of patients with controls. Ultimately, 16,744 GERD patients were matched with 66,976 control participants based on age, sex, income, and region of residence from a total of 514,866 individuals.

The process involved several steps including initial screening, application of inclusion and exclusion criteria, and final matching of patients with controls. Ultimately, 16,744 GERD patients were matched with 66,976 control participants based on age, sex, income, and region of residence from a total of 514,866 individuals (Figure 1).

### 2.5. Covariates

The criteria used to categorize each covariate in the study were defined as follows. Participants were categorized into 10 age groups, divided into 5-year intervals, ranging from 40–44 years to 85 years and older. Income levels were divided into five categories, ranging from class 1 (lowest earnings) to class 5 (highest earnings). Residential regions were classified into 16 areas based on administrative districts and were further grouped into urban and rural categories [28]. We applied the same categorization approach as a previous study for three variables [28]: obesity, smoking, and alcohol drinking. Obesity was classified using BMI (measured in kg/m^2^) [31]. Also, health check-up data included blood pressure, fasting glucose, and cholesterol levels.

The Charlson Comorbidity Index (CCI), ranging from 0 to 29, was used to assess disease burden based on 17 comorbidities, but without including GERD [32]. The CCI scores reflected the severity and number of their comorbid conditions. To minimize the potential confounding effects, comorbidities were adjusted as covariables in analyzing the association of GERD with periodontitis incidence.

### 2.6. Statistical Analyses

Categorical data were expressed as counts and percentages, and continuous data were summarized as means with standard deviations. Standardized differences were used to compare the balance of covariate distribution and general characteristics between the GERD group and the control group, reducing bias due to intergroup imbalance. Covariate balance was evaluated by examining the standardized differences before and after matching, with a difference of less than 0.20, indicating good balance for a particular covariate and effective bias reduction [33].

We used propensity score matching techniques to maintain covariate balance, minimize selection bias, and reduce the impact of potential confounding elements, while preserving heterogeneity between the two cohort groups [34]. The propensity score was generated using multivariable logistic regression based on four baseline covariates: age, sex, income, and residential region. A nearest-neighbor matching algorithm was then applied to pair GERD patients with controls during the periodontitis after the index date in the GERD group compared to the control group, we employed the Cox proportional hazards model on the matched groups. This analysis allowed us to calculate the crude and adjusted hazard ratios (cHRs and aHRs) and their corresponding 95% confidence intervals (CIs). We performed using two models: a crude model and an adjusted model. The adjusted model accounted for potential confounders, specifically obesity, smoking status, alcohol consumption, blood pressure, fasting blood glucose, total cholesterol, and CCI scores. Subgroup analyses were also performed by categorizing all the covariates into subgroups. The study determined crude incidence rates by dividing the number of events by the total person-years of observation, presented as cases per 1000 person-years. The Kaplan–Meier method and log-rank test were then employed to compare the cumulative incidence of periodontitis between the GERD and control groups.

We used SAS version 9.4 (SAS Institute Inc., Cary, NC, USA) and considered a *p*-value of less than 0.05 as statistically significant, based on two-tailed tests for all statistical analyses.

## 3. Results

### 3.1. Demographics

The study analyzed 16,744 individuals with GERD and 66,976 matched controls using data from 2004 to 2019. Participants were carefully matched by age, sex, income, and region, achieving a standardized difference of 0.00, ensuring perfect demographic alignment between groups. Other baseline clinical characteristics showed standardized differences of ≤0.20, indicating minimal variation between the two groups (Table 1).

### 3.2. Association of Occurrence of Periodontitis Between the Group with GERD and the Controls

**Figure 2:**
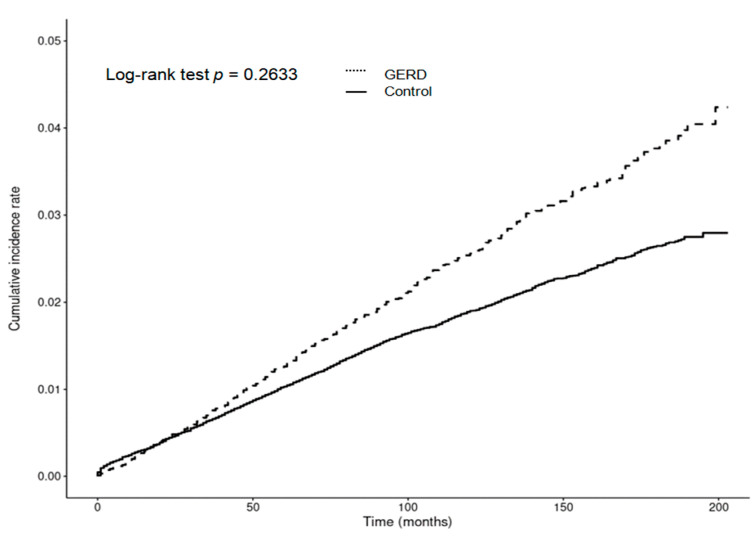
The cumulative risk of developing periodontitis was comparable between individuals with GERD and those without, showing that overall GERD patients did not face an increased cumulative incidence of periodontitis.

The incidence rates of periodontitis were 152 per 1000 person-years in the GERD group and 150 per 1000 person-years in the control group. There were no significant differences in the HRs for developing periodontitis between the GERD and control groups in either the crude (cHR = 1.01, 95% CI = 0.99–1.03, *p* = 0.538) or adjusted models (aHR = 1.00, 95% CI = 0.99–1.02, *p* = 0.625) (Table 2). Additionally, the cumulative incidence rates of periodontitis were similar between the GERD and control groups, as demonstrated by a Kaplan–Meier analysis with a log-rank test, which showed no significant difference in periodontitis risk during the follow-up period (*p* = 0.2633; Figure 2).

### 3.3. Subgroup Analysis

We conducted an in-depth analysis of the relationship between GERD and the incidence of periodontitis by categorizing patients based on several factors, including age, sex, income, place of residence, weight status, smoking and drinking habits, fasting blood glucose levels, CCI scores, and total cholesterol levels.

Our findings revealed notable exceptions in specific subgroups. Participants over the age of 60 and those in lower-income groups had a slightly higher likelihood of developing periodontitis after a GERD diagnosis, with statistically significant results even after full adjustment (aHR = 1.03, 95% CI = 1.00–1.06, *p* = 0.050 for age > 60; aHR = 1.03, 95% CI = 1.01–1.06, *p* = 0.023 for low income).

In contrast, other subgroups, such as sex and region, did not show significant differences in periodontitis risk. Additionally, certain subgroups—such as past/current smokers, those who consumed alcohol less than once a week, individuals with hypertension, those with total cholesterol levels below 200 mg/dL, and those with a CCI score of 0—displayed inconsistent or transient associations in HRs across crude and adjusted models.

## 4. Discussion

Despite the growing body of literature on GERD and its related oral health issues, large-scale nationwide studies assessing the relationship between GERD and the likelihood of subsequent periodontitis using a comprehensive, well-matched national cohort dataset are limited. The association between GERD and the likelihood of developing periodontitis remains controversial. In the present study, we did not find a significant difference in the overall risk of developing periodontitis between GERD patients and controls. By utilizing data from the Korean National Health Screening Cohort, applying propensity score matching for demographic factors, and performing Cox proportional hazards regression analysis to account for confounding variables, we found that GERD was not significantly associated with an increased risk of periodontitis compared to the control group. Additionally, Kaplan–Meier analysis and the log-rank test indicated no significant difference in the cumulative incidence of periodontitis between the GERD and control groups over the 16-year follow-up period.

Our findings align, in part, with a cross-sectional study from Japan, which also found no significant association between GERD and periodontitis after multiple logistic regression analysis [27]. That study, conducted over one year with a small sample of 280 individuals attending a health center for medical checkups, examined the relationship between GERD and periodontitis [27]. However, the cohort was demographically imbalanced, with 68% male and 32% female participants [27]. It also lacked consideration of socioeconomic status; although, the authors assumed most participants were middle class but without concrete evidence [27]. Furthermore, the study did not account for comorbidities [27]. As a small, single-center cross-sectional study, it may not adequately represent the broader population [27], hindering the generalization of the connection between GERD and incident periodontitis.

In our study, based on the largest representative population sample (n = 83,720) compared to previous research, our findings differ from those of two smaller Korean population-based clinical studies (n = 560 and n = 731, respectively) [25,26], a small study from India (n = 60) [35], a study from Hungary (n = 116) [36], and a large nationwide cohort study from Taiwan (n = 40,250) [24]. Most of these studies reported a modest association between GERD and periodontitis [24,26,35,36]. The smaller studies were based on experiences from single tertiary hospitals and generally had short follow-up periods [24,25,26,36]. One of the Korean studies (n = 560) reported a 2.883-fold increased likelihood of periodontitis (95% CI, 1.775–4.682), identifying GERD as an independent risk factor for periodontitis [25]. However, the small sample size and lack of follow-up data may have overstated the study’s power to detect associations, limiting its ability to fully assess periodontitis development in GERD patients. The second Korean study focused on obstetric patients during pregnancy, investigating the relationships between preterm birth, GERD, and periodontitis to identify predictors of preterm labor [26], though the findings may be inconsistent. While some studies have reported a stronger association between preterm birth and GERD than with periodontitis [37], a recent Mendelian randomization study found no evidence supporting a causal relationship between periodontitis and preterm birth, nor vice versa [38]. Given the potential for pregnancy to increase the frequency of transient GERD, it is likely a confounding factor in the observed associations. None of these studies employed a matching process between the study and control groups [24,25,26,36], leading to imbalances in demographic data, socioeconomic status, and comorbid conditions, which may have compromised the validity of their findings.

Our study results also diverge from those of a large Taiwanese nationwide cohort study [24], which utilized the Taiwan National Health Insurance Research Database, a universal healthcare system similar to Korea’s, covering the majority of the population. In that study, Li et al. [24] conducted a 1:1 propensity score-matched analysis of 20,125 GERD patients and 20,125 controls, finding a modest association between GERD and periodontitis (aHR 1.36; 95% CI, 1.28–1.45) over an approximately 10-year follow-up. However, their analysis only adjusted for age, sex, and certain comorbidities, without accounting for socioeconomic or lifestyle factors, which are significant contributors to the development of GERD [4,5,6]. This may have introduced potential confounding variables. In contrast, our study achieved a balanced distribution of demographic, socioeconomic, lifestyle, and health-related factors by matching 16,744 GERD patients with 66,976 non-GERD participants at a 1:4 ratio. This ratio was chosen for two key reasons: first, 1:1 matching risked significant data loss from the GERD group when propensity scores differed between the GERD and control groups. Second, although 1:2 matching offers some advantages over 1:1, research suggests that matching ratios beyond 1:5 provide minimal improvements in test power, with no significant statistical gain beyond a 1:4 ratio [39]. To further minimize confounding, we employed a methodologically robust design using nationwide data, adjusting for a broad range of potential confounders, including demographic, socioeconomic, lifestyle, and comorbid factors, such as age, sex, income, residential area, obesity status, smoking habits, alcohol consumption, systolic and diastolic blood pressure, fasting blood glucose, total cholesterol, and CCI scores. This comprehensive approach allowed for a more accurate exploration of the relationship between GERD and periodontitis. While we reproduced the conclusion of no overall increased risk of periodontitis following GERD, a subset of the GERD group under certain conditions (such as individuals aged over 60 years old and those in low-income brackets) showed a slightly elevated likelihood of developing periodontitis during the 16-year long-term follow-up analysis.

Subgroup analyses revealed that individuals over 60 years old and those in lower-income brackets exhibited a slightly increased risk of developing periodontitis following GERD; although, the overall GERD group did not show a significantly higher risk compared to the control group. Overall, our results suggest that the relationship between GERD and periodontitis is multifaceted, with the risk of developing periodontitis varying based on age and socioeconomic factors. These findings point to the need for a nuanced approach to managing GERD, particularly in older adults and lower-income populations, to mitigate the risk of periodontitis. GERD is influenced by various risk factors, including age, sex, race, smoking, alcohol intake, stress, diet, obesity, low income, physical inactivity, family history, and genetic predispositions, like polymorphisms [4,40,41]. Its prevalence increases with age, with a pooled prevalence ratio of 1.17 (95% CI, 1.11–1.24) in individuals aged 60 and older compared to those aged 18–34 [3] and a meta-analysis confirming a significantly higher prevalence in those aged 50 and above [42].

Similarly, periodontitis incidence tends to rise with age, particularly affecting males, individuals with lower education levels and income, and those in rural areas [43,44,45]. Older adults are especially vulnerable to periodontal disease due to factors such as decreased saliva production, a higher prevalence of chronic diseases, and potential delays in seeking medical or dental care [46]. Socioeconomic disparities may play a significant role in the severity of periodontal disease, with lower education and income strongly associated with worse outcomes [47]. Studies consistently show an inverse relationship between periodontal disease and socioeconomic status, even when adjusting for age and sex [47,48].

These shared risk factors between GERD and periodontitis may suggest that age and socioeconomic status may influence the likelihood of periodontitis development in GERD patients. GERD’s exposure of the oral cavity to gastric acids can damage oral tissues, making them more prone to infection and inflammation [25]. Chronic inflammation, common in both GERD and periodontitis, may further exacerbate periodontal issues through systemic inflammatory pathways [49]. Given these findings, it is important to monitor oral health in older adults with GERD to mitigate periodontitis risk. Additionally, lower-income individuals, with less access to healthcare and dental services, face greater challenges in managing periodontal disease progression [50]. This may underscore the need for public health interventions to improve healthcare access for economically disadvantaged groups.

A Mendelian randomization study using genome-wide association data from individuals of European ancestry found that GERD increased the risk of gingivitis and periodontal disease (odds ratio = 1.166, 95% CI, 1.046–1.190, *p* = 0.001) [41]. The differences between these findings and our study may be attributed to ethnic variations. Periodontitis is more common and severe in American and European adults compared to East Asian adults, with at least 46% of individuals over 30 years old affected and 10% experiencing severe periodontitis [12,51]. Additionally, GERD is more prevalent in American and European populations than in East Asian populations [2,3], which may partly explain the discrepancies in prevalence and outcomes between the studies.

The present study exhibited several restrictions. Based on its retrospective and observational structure, we cannot establish a definitive causal relationship between GERD and periodontitis. Additionally, we did not explore the fundamental processes that might explain the relationship between these two issues. Our study was limited to Korean citizens over the age of 40 and based on diagnosis codes from Korean health insurance data, which may have introduced unaccounted confounding factors and reduced the generalizability of the findings to other populations and age groups. Third, the sample cohort data from the Korean National Health Insurance Service only included the ICD-10 code for ‘chronic periodontitis’, without details on disease severity or stages. This limitation prevents us from applying the updated classification system based on stages and grades, as recommended by the 2017 World Workshop on Periodontal and Peri-Implant Diseases [18]. We acknowledge this limitation and suggest caution when interpreting the findings. Unfortunately, this study does not include detailed information regarding the treatment outcomes for periodontitis, and our access to the KNHIS-HSC data has since expired. As a result, we were unable to assess the impact of specific treatments, such as surgical versus nonsurgical interventions or adjunctive therapies, on the overall conclusions. This limitation represents a potential source of bias and should be considered when interpreting our findings.

Nonetheless, this study sought to evaluate the relationship between GERD and the risk of developing periodontitis using a robust, carefully matched nationwide cohort dataset representative of the adult population in Korea. The study’s strength and reliability were bolstered by utilizing a nationally validated cohort database, which enabled matching patients with control subjects using propensity scores. This method reduced selection bias and reinforced the validity of the findings [52]. Moreover, the use of the KNHIS-HSC database provided comprehensive access to each participant’s medical history from healthcare facilities across the nation, further improving the generalizability and precision of the results. A key strength of the study was the thorough control for potential confounding components, including social and economic determinants (including place of residence and income), lifestyle determinants (including obesity, alcohol consumption, fasting blood glucose, smoking, blood pressure level, and total cholesterol), and prior health conditions. This thorough refinement process enhanced the accuracy and reliability of the conclusions. At last, the study’s 16-year follow-up period—one of the longest in research on the relationship between GERD and periodontitis—offered a significant benefit in reproducing the absence of a consistent relationship between these two conditions over an extended timespan.

## 5. Conclusions

This study did not establish a definitive overall association between GERD and the development of periodontitis. However, subgroup analyses identified a slightly increased risk of periodontitis in individuals over 60 years old and those in lower-income brackets following GERD diagnosis. These findings may suggest the need for preventive measures and closer monitoring of oral health in these vulnerable populations. Additionally, addressing socioeconomic barriers to healthcare access is crucial. Future research should explore the mechanistic links between GERD and periodontitis and include diverse populations to enhance understanding and inform integrated healthcare strategies.

## Figures and Tables

**Table 1:** Demographic information of participants.

Characteristics	Total Participants		
	GERD	Control	Standardized Difference
Total number			
Age (years old) (n, %)			0.00
40–44	746 (4.46)	2984 (4.46)	
45–49	3393 (20.26)	13,572 (20.26)	
50–54	2964 (17.70)	11,856 (17.70)	
55–59	2373 (14.17)	9492 (14.17)	
60–64	2107 (12.58)	8428 (12.58)	
65–69	2239 (13.37)	8956 (13.37)	
70–74	1697 (10.13)	6788 (10.13)	
75–79	877 (5.24)	3508 (5.24)	
80–84	329 (1.96)	1316 (1.96)	
85+	19 (0.11)	76 (0.11)	
Sex (n, %)			0.00
Male	7908 (47.23)	31,632 (47.23)	
Female	8836 (52.77)	35,344 (52.77)	
Income (n, %)			0.00
1 (lowest)	2732 (16.32)	10,928 (16.32)	
2	2369 (14.15)	9476 (14.15)	
3	2640 (15.77)	10,560 (15.77)	
4	3537 (21.12)	14,148 (21.12)	
5 (highest)	5466 (32.64)	21,864 (32.64)	
Region of residence (n, %)			0.00
Urban	6942 (41.46)	27,768 (41.46)	
Rural	9802 (58.54)	39,208 (58.54)	
Obesity † (n, %)			0.08
Underweight	452 (2.70)	1861 (2.78)	
Normal	6230 (37.21)	24,112 (36.00)	
Overweight	4560 (27.23)	17,789 (26.56)	
Obese I	5072 (30.29)	21,105 (31.51)	
Obese II	430 (2.57)	2109 (3.15)	
Smoking status (n, %)			0.06
Nonsmoker	12,187 (72.78)	48,072 (71.77)	
Past smoker	1426 (8.52)	5098 (7.61)	
Current smoker	3131 (18.70)	13,806 (20.61)	
Alcohol consumption (n, %)			0.02
<1 time a week	12,733 (76.05)	50,249 (75.03)	
≥1 time a week	4011 (23.95)	16,727 (24.97)	
Systolic blood pressure (mean, SD)	126.28 (17.29)	128.16 (18.33)	0.11
Diastolic blood pressure (mean, SD)	78.36 (10.95)	79.50 (11.44)	0.10
Fasting blood glucose (mean, SD)	97.59 (28.17)	98.81 (30.75)	0.04
Total cholesterol (mean, SD)	199.43 (38.13)	199.79 (37.89)	0.01
CCI score (mean, SD)	1.22 (1.88)	1.05 (1.75)	0.10
Periodontitis (n, %)	13,251 (79.14)	52,626 (78.57)	0.01

Abbreviations: GERD, gastroesophageal reflux disease; CCI, Charlson Comorbidity Index; SD, standard deviation. † Obesity (BMI, body mass index, kg/m^2^) was categorized as <18.5 (underweight), ≥18.5 to <23 (normal), ≥23 to <25 (overweight), ≥25 to <30 (obese I), and ≥30 (obese II).

**Table 2:** Crude and overlap propensity score weighted hazard ratios (95% confidence interval) of GERD for periodontitis.

	N of Periodontitis/ N of Total (%)	Follow-Up Duration (PY)	IR per 1000 (PY)	IRD (95% CI)	Hazard Ratios for Periodontitis			
					Crude †	*p*	Adjusted †‡	*p*
Total participants								
GERD	13,251/16,744 (79.14)	87,214	152	2.00 (−0.73–5.02)	1.01 (0.99–1.03)	0.538	1.00 (0.99–1.02)	0.625
Control	52,626/66,976 (78.57)	351,333	150		1		1	
Age < 60 years old								
GERD	8290/9476 (87.48)	49,933	166	−2.00 (−5.70–2.33)	0.99 (0.97–1.01)	0.450	0.99 (0.97–1.01)	0.413
Control	33,176/37,904 (87.53)	197,824	168		1		1	
Age ≥ 60 years old								
GERD	4961/7268 (68.26)	37,281	133	6.00 (2.32–10.42)	1.03 (1.00–1.07)	0.042 *	1.03 (1.00–1.06)	0.050 *
Control	19,450/29,072 (66.90)	153,509	127		1		1	
Male								
GERD	6331/7908 (80.06)	38,234	166	5.00 (0.05–9.06)	1.02 (0.99–1.05)	0.225	1.01 (0.99–1.04)	0.299
Control	25,028/31,632 (79.12)	155,424	161		1		1	
Female								
GERD	6920/8836 (78.32)	48,980	141	0.00 (−3.31–4.13)	1.00 (0.97–1.02)	0.767	1.00 (0.97–1.02)	0.751
Control	27,598/35,344 (78.08)	195,909	141					
Low-income group								
GERD	5918/7741 (76.45)	40,796	145	6.00 (2.48–10.56)	1.04 (1.01–1.07)	0.017 *	1.03 (1.01–1.06)	0.023 *
Control	23,379/30,964 (75.50)	168,749	139		1		1	
High-income group								
GERD	7333/9003 (81.45)	46,418	158	−2.00 (−6.28–1.87)	0.98 (0.96–1.01)	0.209	0.98 (0.96–1.01)	0.186
Control	29,247/36,012 (81.21)	182,584	160		1		1	
Urban resident								
GERD	5804/6942 (83.61)	34,684	167	2.00 (−2.90–6.68)	1.01 (0.98–1.03)	0.743	1.00 (0.97–1.03)	0.894
Control	22,997/27,768 (82.82)	138,995	165		1		1	
Rural resident								
GERD	7447/9802 (75.97)	52,530	142	2.00 (−1.34–5.80)	1.01 (0.98–1.03)	0.594	1.01 (0.98–1.03)	0.563
Control	29,629/39,208 (75.57)	212,338	140		1		1	
Underweight								
GERD	256/452 (56.64)	2414	106	2.00 (−11.79–16.79)	1.01 (0.89–1.16)	0.836	1.03 (0.90–1.18)	0.666
Control	1068/1861 (57.39)	10,314	104		1		1	
Normal weight								
GERD	4800/6230 (77.05)	32,943	146	4.00 (−0.87–8.27)	1.02 (0.99–1.06)	0.160	1.01 (0.98–1.05)	0.360
Control	18,348/24,112 (76.09)	129,205	142		1		1	
Overweight								
GERD	3674/4560 (80.57)	23,342	157	2.00 (−3.39–7.93)	1.01 (0.97–1.05)	0.607	1.01 (0.97–1.05)	0.628
Control	14,337/17,789 (80.59)	92,421	155		1		1	
Obese								
GERD	4521/5502 (82.17)	28,515	159	1.00 (−4.66–5.61)	1.00 (0.97–1.03)	0.949	1.00 (0.96–1.03)	0.840
Control	18,873/23,214 (81.30)	119,393	158		1		1	
Nonsmoker								
GERD	9634/12,187 (79.05)	65,430	147.00	1.00 (−1.62–4.93)	1.01 (0.99–1.03)	0.467	0.98 (0.96–1.01)	0.154
Control	37,811/48,072 (78.65)	259,720	146.00		1		1	
Past and current smoker								
GERD	3617/4557 (79.37)	21,784	166.00	4.00 (−1.63–10.28)	1.02 (0.98–1.06)	0.258	0.96 (0.93–1.00)	0.028 *
Control	14,815/18,904 (78.37)	91,613	162.00		1		1	
Alcohol consumption < 1 time a week								
GERD	9967/12,733 (78.28)	67,780	147.00	1.00 (−2.50–3.95)	1.00 (0.98–1.02)	0.868	0.97 (0.95–0.99)	0.013 *
Control	39,210/50,249 (78.03)	267,968	146.00		1		1	
Alcohol consumption ≥ 1 time a week								
c	3284/4011 (81.87)	19,434	169.00	8.00 (1.76–14.34)	1.05 (1.01–1.09)	0.021 *	0.99 (0.95–1.03)	0.643
Control	13,416/16,727 (80.21)	83,365	161.00		1		1	
SBP < 120 mmHg and DBP < 80 mmHg								
GERD	3789/4624 (81.94)	23,653	160.00	0.00 (−5.25–6.24)	1.00 (0.97–1.04)	0.895	0.99 (0.96–1.03)	0.753
Control	13,861/16,758 (82.71)	86,796	160.00		1		1	
SBP ≥ 120 mmHg or DBP ≥ 80 mmHg								
GERD	9462/12,120 (78.07)	63,561	149.00	2.00 (−0.99–5.65)	1.01 (0.99–1.03)	0.311	0.97 (0.95–0.99)	0.010 *
Control	38,765/50,218 (77.19)	264,537	147.00		1		1	
Fasting blood glucose < 100 mg/dL								
GERD	9040/11,305 (79.96)	59,679	151.00	0.00 (−2.93–4.05)	1.00 (0.98–1.02)	0.915	0.98 (0.96–1.00)	0.065
Control	35,788/44,769 (79.94)	237,139	151.00		1		1	
Fasting blood glucose ≥ 100 mg/dL								
GERD	4211/5439 (77.42)	27,535	153.00	6.00 (0.41–10.55)	1.03 (1.00–1.07)	0.075	0.97 (0.94–1.01)	0.115
Control	16,838/22,207 (75.82)	114,194	147.00		1		1	
Total cholesterol < 200 mg/dL								
GERD	6908/8806 (78.45)	45,768	151.00	1.00 (−2.98–4.96)	1.00 (0.98–1.03)	0.776	0.97 (0.95–1.00)	0.036 *
Control	27,487/35,101 (78.31)	183,316	150.00		1		1	
Total cholesterol ≥ 200 mg/dL								
GERD	6343/7938 (79.91)	41,446	153.00	3.00 (−0.75–7.59)	1.02 (0.99–1.05)	0.185	0.98 (0.96–1.01)	0.209
Control	25,139/31,875 (78.87)	168,017	150.00		1		1	
CCI scores = 0								
GERD	7686/8970 (85.69)	47,930	160.00	3.00 (−0.43–7.44)	1.02 (1.00–1.05)	0.084	0.97 (0.95–1.00)	0.020 *
Control	33,327/39,628 (84.10)	212,474	157.00		1		1	
CCI scores = 1								
GERD	2374/2988 (79.45)	15,901	149.00	5.00 (−1.82–11.61)	1.03 (0.98–1.08)	0.211	0.97 (0.93–1.02)	0.200
Control	8107/10,632 (76.25)	56,141	144.00		1		1	
CCI scores ≥ 2								
GERD	3191/4786 (66.67)	23,383	136.00	1.00 (−4.18–6.51)	1.00 (0.96–1.04)	0.965	1.00 (0.96–1.04)	0.963
Control	11,192/16,716 (66.95)	82,718	135.00		1		1	

Abbreviations: GERD, gastroesophageal reflux disease; SBP, systolic blood pressure; DBP, diastolic blood pressure; CCI, Charlson Comorbidity Index; PY, person-years; IR, incidence rate; IRD, incidence rate difference; CI, confidence interval. *, significance at *p* < 0.05. †, models were stratified by age, sex, income, and region of residence; ‡, adjusted for CCI scores.

## Data Availability

All data are available from the database of National Health Insurance Sharing Service (NHISS) https://nhiss.nhis.or.kr/ (accessed on 1 March 2024). NHISS allows access to all of these data for the any researcher who promises to follow the research ethics at some processing charge. If you want to access the data in this article, you can download it from the website after promising to follow the research ethics.
